# A window into the brain mechanisms associated with noise sensitivity

**DOI:** 10.1038/srep39236

**Published:** 2016-12-15

**Authors:** Marina Kliuchko, Marja Heinonen-Guzejev, Peter Vuust, Mari Tervaniemi, Elvira Brattico

**Affiliations:** 1 Cognitive Brain Research Unit, Institute of Behavioural Sciences, University of Helsinki, Helsinki, FI-00014, Finland; 2 BioMag Laboratory, HUS Medical Imaging Center, University of Helsinki and Helsinki University Hospital, Helsinki, FI-00029, Finland; 3 Department of Public Health, University of Helsinki, Helsinki, FI-00014, Finland; 4 Center for Music in the Brain (MIB), Department of Clinical Medicine, Aarhus University, Aarhus, DK-8000, Denmark; 5 Cicero Learning, University of Helsinki, Helsinki, FI-00014, Finland

## Abstract

Noise sensitive individuals are more likely to experience negative emotions from unwanted sounds and they show greater susceptibility to adverse effects of noise on health. Noise sensitivity does not originate from dysfunctions of the peripheral auditory system, and it is thus far unknown whether and how it relates to abnormalities of auditory processing in the central nervous system. We conducted a combined electroencephalography and magnetoencephalography (M/EEG) study to measure neural sound feature processing in the central auditory system in relation to the individual noise sensitivity. Our results show that high noise sensitivity is associated with altered sound feature encoding and attenuated discrimination of sound noisiness in the auditory cortex. This finding makes a step towards objective measures of noise sensitivity instead of self-evaluation questionnaires and the development of strategies to prevent negative effects of noise on the susceptible population.

Noise is ubiquitous in the modern world. While a mild amount of noise can even be beneficial to periphery neurons^1^, constant loud noise in the environment leads to damage, not only to the auditory system (peripheral^2^ and central^3^,^4^), but also to other body organs. Cardiovascular diseases such as hypertension, coronary heart disease, and myocardial infarctions are more frequent in individuals exposed to environmental^5^ or occupational noise^6^. Remarkably, the increase in risk for non-auditory diseases associated with noise exposure is highly variable. This individual variability has been isolated as a stable trait, termed noise sensitivity (NS)^7^,^8^. Noise sensitive individuals seem to be more susceptible than non-sensitive individuals to the adverse effects of noise such as sleep disturbance^9^, impaired cognitive performance^10^, and cardiovascular disease^11^. The current evaluation of NS is, however, limited to self-evaluation questionnaires and no objective measures have yet been put forward.

In the context of NS, noise is referred to as unwanted sound. In essence, a sound can be subjectively perceived as noise regardless of its actual level^11^,^12^,^13^. In modern society, noise pollution is unfortunately very common and even if disturbing to all individuals^3^,^14^, it is particularly disturbing to noise sensitive individuals. According to the definition, NS is described as physiological and psychological internal states, which increase the degree of reactivity to noise in general^15^. The causes of NS are not understood. NS is independent of noise exposure, such as living in a noisy environment^16^,^17^, and it is nonspecific to noise sources^15^. NS is not directly related to sound intensity, but it predicts noise annoyance that is known to increase with a sound intensity level. NS is considered to be a factor that moderates the relationship between noise and the annoyance that it induces^7^,^16^.

NS is a common trait that concerns about 20–40%^18^ of the non-clinical population, and the prevalence of high NS is estimated to be between 12% to 15%^16^,^19^. NS aggregates in families, meaning a higher frequency of NS in first-degree relatives compared to the general population, with a heritability estimate of 36%^20^.

NS is not a synonym for hyperacusis, which is also a common symptom in patients with tinnitus, William’s syndrome, autism, and other neurologic diseases^21^. Hyperacusis is a loudness-related hypersensitivity to sounds that causes an experience of discomfort at lower loudness levels than normally. It encompasses a wide range of reactions to sound, which can be excessive loudness annoyance, fear or pain^22^. The current understanding is that hyperacusis results from the malfunction of the central auditory pathways and their connections within the central nervous system rather than the dysfunction at the peripheral level^23^, whereas the mechanisms underlying NS are less well-understood.

Research brings no strong evidence for the relation of NS to performance in intensity discrimination or hearing acuity between individuals with low and high NS^13^. This suggests that NS may relate to functional changes in the central nervous system. However, as psychometric and psychoacoustic approaches have been prevalent in NS research, the neurological underpinnings of NS are undetermined^24^. Nevertheless, only by understanding the neural mechanisms of NS is it possible to validate the existing knowledge of the NS phenomenon, and to attempt to design appropriate preventive and intervention strategies to avoid adverse effects of noise on sensitive individuals.

In this study, we addressed the neural mechanisms of the sound processing underlying NS using combined electro- and magnetoencephalography (EEG/MEG). We presented 71 subjects with a multifeature mismatch negativity (MMN) paradigm (Fig. 1), where a noise feature was embedded in an auditory complex context along with five other deviant features. High frequency of occurrence of each deviant makes it possible to measure discrimination abilities in a short time, as compared to classical oddball paradigms. Yet, MMN responses obtained with the multifeature paradigm yield comparable parameters to those observed with a classical oddball paradigm^25^,^26^. Furthermore, the complex structure of the multifeature MMN paradigm adds ecological validity to the stimuli. In recent years, the multifeature paradigm has been successfully adopted for obtaining neurophysiological measures of auditory discrimination in special populations, such as cochlear implantees^27^,^28^, depressed patients^29^, patients with panic disorder^30^ and individuals with different skills in music^31^,^32^,^33^. Moreover, the linguistic multifeature paradigm is increasingly used in speech perception research and shown to be efficient in recording auditory discrimination profiles even in toddlers^34^.

By using the multifeature paradigm in our study, we probed the efficiency of sensory processing in the auditory cortex as reflected by the P1 component of an event-related potential (ERP), and the accuracy of automatic sound feature discrimination as reflected by MMN. NS has not been previously researched with MMN. Based on previous behavioral and neurophysiological findings, we hypothesize that NS may be related to the processing of noise. We expect to observe this as differences in parameters of MMN to the noise deviant between individuals with high and low NS. Additionally, we predict an effect of NS on the P1 response, because the differences in early ERP components between noise sensitive and non-sensitive individuals have previously been indicated. Evidence for this comes from scarce electrophysiological research on NS^35^ and annoyance caused by an unpleasant sound^36^.

## Results

### Noise sensitivity

The mean NS score in the sample was 80.5 (SD = 17.4). NS did not differ between male and female subjects (F_1,70_ = 2.47, *P* = 0.121). In order to make group-wise comparisons, a tertile split was performed on NS scores resulting in three groups of subjects exhibiting low (N = 23, M = 62.2, SD = 6.5), medium (N = 23, M = 78.1, SD = 4.2) and high (N = 25, M = 99.5, SD = 11.0) NS.

NS did not correlate with the depression score on HADS (r = −0.088, *P* = 0.49) and there were no significant differences in depression score between the three NS groups (F_1,62_ = 1.28, *P* = 0.29).

### EEG/MEG data description

P1 was measured from an ERP to standard stimuli. Parameters of P1 responses are presented in Table 1.

According to the results of the statistical analysis, significant MMN responses were elicited by all deviants (for the measured *P* values see Supplementary Table S1). Positive MMN reversal at inferior temporal electrodes was registered for all deviants, confirming that the response is MMN and not e.g. N2b, which is a component that occurs at a similar latency and indicates an attention switch^37^. MMN waveforms are illustrated in Figure S1 of the Supplementary information. The highest amplitude of MMN was observed for the location deviant (P < 0.001). The lowest amplitude of MMN was registered to the intensity deviant (P ≤ 0.045 for all comparisons).

In the MEG data, we observed the largest MMNm responses elicited by the location and slide deviants (Slide-MMNm vs. Location-MMNm: *P* = 0.842; all other comparisons: *P* < 0.001), whereas the intensity MMNm was the smallest of all (*p* < 0.0001). MMNm waveforms are presented in Figure S2 of the Supplementary information.

### Group comparisons: EEG data

ANCOVA on P1 amplitude at Fz electrode with NS groups (low, medium and high) as between-subjects factors revealed a significant main effect of Group (F_2,61_ = 7.71, *P* = 0.001, ηp2 = 0.202). The P1 was the highest in the low NS group as compared to the medium and high NS groups (*P* = 0.003 and *P* = 0.001, respectively; Fig. 2). Neither Years of Musical Training nor Age showed significant effects (*P* = 0.051 and *P* = 0.510, respectively).

The MMN amplitudes were compared between the three NS groups by repeated measures ANOVA with Deviant as the within-subjects factor. We found a significant main effect of Group (F_2,61_ = 6.50, *P* = 0.003, ηp2 = 0.176). Post hoc comparison showed a higher amplitude of MMN in the low NS group than in the medium (*P* = 0.001) and high (*P* = 0.028) NS groups. We observed a significant effect for the Years of Musical Training covariate (F_1,61_ = 4.04, *P* = 0.049, ηp2 = 0.062), but not for Age (*P* = 0.110).

The effect of Deviant factor was significant (F_5,305_ = 4.01, *P* = 0.004, ηp2 = 0.062) suggesting that the amplitude of MMN varied depending on the type of deviant. We used separate ANCOVAs to determine the effect of NS on discrimination of each stimulus type. To account for multiple testing (N = 6), we applied the Bonferroni correction, whereupon only *P* values below 0.008 were considered significant. We found that the amplitude of MMN to noise varied between the groups (main effect of Group: F_2,61_ = 6.14, *P* = 0.004, ηp2 = 0.168). According to post hoc testing, the low NS group had stronger MMN to the noise deviant than medium and high NS groups (*P* = 0.002 and *P* = 0.010, respectively; Fig. 2). Years of Musical Training and Age did not have significant effects on MMN to the noise deviant (*P* > 0.050 for both covariates). No other MMN amplitudes were found to significantly differ between NS groups or to survive the correction for multiple testing. However, when uncorrected, there was an effect of NS on the amplitude of MMN to the location deviant (P = 0.027; post hoc comparison of low NS group vs. medium NS group: P = 0.009). Statistical details of all performed ANCOVAs are presented in Table S2 of the Supplementary information.

Analysis of the P1 and the MMN latencies did not reveal any significant differences between NS groups.

### Group comparisons: MEG data

Analysis of MMNm (MEG equivalent of MMN) replicated the EEG results on the relationship between NS and MMN showing the main effect of Group (F_2,66_ = 3.36, *P* = 0.041, ηp2 = 0.092) that was due to lower MMNm amplitudes in the high NS group than in the low NS group (LSD post hoc test: *P* = 0.012). The effect of Deviant was also significant (F_5,330_ = 3.88, *P* = 0.009, ηp2 = 0.055). The post hoc LSD test showed that all deviants were different in the strength of evoked responses except for location vs. slide (P = 0.824), pitch vs. rhythm (P = 0.488) and pitch vs. noise (P = 0.061). We also observed an effect of Age covariate (F_2,66_ = 4.89, *P* = 0.031, ηp2 = 0.069) but not of Years of Musical Training (P = 0.060). Both of the covariates showed a significant interaction with the type of deviation (Deviant × Age: F_5,330_ = 9.10, *P* = 0.001, ηp2 = 0.121; Deviant x Years of Musical Training: F_5,330_ = 5.36, *P* < 0.0001, ηp2 = 0.075). Assuming that older subjects may have continued with more years of musical practice after finishing their training, we think that musical experience could explain the observed effects of age. To test this assumption, we performed a correlational analysis and found that Age was significantly correlated with Years of Musical Experience (r = 0.270, P = 0.023) whereas the correlation with Years of Musical Training was not significant (r = 0.161, P = 0.179). We left a further discussion on the effects of musical practice on MMN out of the scope of this paper as it is in the focus of another study that will be reported elsewhere.

Further, we performed separate repeated measures ANOVAs on each type of the deviant with NS groups (low, medium and high) as between-subjects factors and MMNm amplitude at eight regions of interest (ROIs) as within-subjects factors. Statistical results for each ANCOVA are reported in Table S3 of the Supplementary information. None of the P values survived correction for multiple comparisons. However, prior to the correction, we observed a significant main effect of Group (F_2,66_ = 3.82, *P*_*uncorr.*_ = 0.027, ηp2 = 0.104) on MMNm to the noise deviant. According to LSD post hoc analysis, noise MMNm was significantly smaller in the high NS group than in the low NS group (*P* = 0.007; Fig. 3). No effects of Years of Musical Training (*P* = 0.237) or Age (*P* = 0.058) were observed.

We did not find significant effects of NS group on MMNm to any other deviant (ANCOVA results are reported in Table S3 of the Supplementary information).

## Discussion

Using combined EEG/MEG, we for the first time probed neural responses of the central auditory system to sound feature changes in individuals with NS. We used a multifeature MMN paradigm, which allowed us to test auditory processing of sound variations in an auditory complex music-like context, thus resembling ecologically-valid sound processing. We embedded a deviant with increased noisiness into the paradigm to test the efficiency of automatic processing and discrimination of this sound feature in noise sensitive individuals. Our results showed that NS is associated with functional alternation of auditory stimulus encoding and discrimination of noisy sounds. First, we observed a diminished P1 component of the ERP in subjects with high NS as compared to subjects with low NS. Second, noise sensitive subjects had generally smaller MMN amplitude than non-sensitive subjects. Furthermore, we found evidence for specific attenuation of MMN and MMNm to the noise feature, out of all other features, in subjects with high NS as compared to the low NS group. Taken together, these findings suggest that central mechanisms of auditory processing and discrimination are involved and, specifically, affected in NS.

P1 is thought to reflect the process of sensory gating, which is the ability of the central nervous system to actively inhibit the response to repetitive stimuli (gating out) and increase responses to stimuli with novel features (gating in)^38^. Impaired sensory gating is typical of schizophrenics, and is implicated in attention deficits and perceptual hypersensitivity to sounds in these patients. Shepherd and collegues^35^ were the first to test whether filtering mechanisms are compromised in NS as well. Using a paired-click paradigm, they found that differences in sensory gating might exist between NS extremities: in an auditory attention condition, noise sensitive individuals showed lower sensory gating than the non-sensitive group. In the passive condition, the sensory gating was stronger in both groups than in the attend condition, but the between-groups difference was not significant. Our results, furthermore, suggest that NS may be related to the altered mechanism of filtering of auditory input in the brain. P1 is known to be diminished for a repetitive standard sound and increased after the presentation of a deviant sound, thus reflecting the process of detecting a sound and gating it in^39^. Unlike in odd-ball settings, in our paradigm, the standard stimuli constantly varied in frequency and there were no identical sounds presented consecutively. We would expect that with our paradigm the P1 should not be largely suppressed since the new auditory information is delivered with every sound. However, in the high NS group P1 was smaller than in the low NS group, suggesting an attenuated capacity for processing incoming sound features in a variable sound stream. However, the different methodologies used in our study and previous research on sensory gating^35^,^39^ limits the comparison of the findings.

The attenuated encoding of auditory sensory input, reflected in the amplitude of the P1 response in noise sensitive subjects, was followed by modulated sound representation forming, as was found in smaller MMN and MMNm in the high NS group. Smaller amplitudes of MMN/MMNm suggest less effective automatic detection of changes in the incoming auditory information. Attenuation of both obligatory P1 response and MMN suggests that there are several steps of pre-attentive central auditory processing that are associated with NS. Further investigation of MMN to different sound feature changes revealed that out of six features tested, the noise sensitive individuals appeared to be compromised in discrimination of sounds with increased noisiness. The MEG counterpart of MMN elicited by the noise deviant showed a gradual change in noise discrimination from the low NS group to the high NS group, though this effect was not significant after the correction of P-value. Attenuated MMN to the noise deviant could indicate a smaller perceptual difference between noise levels of standard and deviant sounds in the high NS group.

In recent years perception has been considered in the frame of predictive coding theory, which suggests that perception is organized in a hierarchical manner^40^,^41^,^42^. In this concept, MMN is proposed to be not only a deviance detection component but to reflect an error that occurs when anticipated auditory information does not fit the actual sensory input. The prediction error is small when the sensed information accurately meets the model or when the prediction is weak and uncertainty of expectations is high. It seems that the latter scenario could explain the patterns of automatic auditory processing obtained in our study. Perhaps individuals with high NS were unable to build a precise top-down prediction of sensory input due to an inaccurate encoding of sound features, suggested by suppressed P1. In that case, a high uncertainty of their predictions of perceived auditory information could explain the low MMN amplitude in the high NS group.

Until now, MMN has not been used to study NS but it has been researched in such sound hypersensitivity conditions as tinnitus and hyperacusis. Weisz and colleagues^43^ demonstrated abnormal MMN responses to frequencies at the lesion-edge in tinnitus sufferers as compared to normal hearing controls. MMN responses, and their source locations, were negatively related to emotional-cognitive distress caused by tinnitus. In the study by Boucher *et al*.^44^, no significant effect on P2 or MMN amplitude was found in hyperacusis caused by insular lesion. However, differences in the methodologies of the aforementioned studies and the current work do not allow for a conclusion with regard to whether sound hypersensitivity, as a mutual symptom in tinnitus and hyperacusis, could have shared underlying mechanisms with NS phenomena.

In some studies NS scores have been considerably higher in the depressed patients compared to the control subjects^7^ and an association with the neurotic end of the spectrum of depressive illness has been found^45^. However, there has been no evidence to support an association between NS and major depression in particular^7^, nor was it found in the current data sample. It should be noted, though, that the subjects participating in our study were healthy without diagnosed depression and their mean score on a test for self-evaluation suggested only minimal depressive symptoms. Thus, relatively small variation in their depression scores could actually be the cause of the lack of correlation between depression scores and NS in these results.

NS has been associated with the stronger evaluation of sound unpleasantness^13^, and self-reported hearing disabilities^46^. Some of the existing views on the origin of NS suggest that it might be related to a generally increased sensitivity to stimuli of different modalities, and inclination of noise sensitive individuals to report stronger negative reactions towards them^47^,^48^. A recent epidemiological study reports that up to 88% of individuals with high noise sensitivity self-declare at least one other environmental sensitivity^17^. However, research does not always confirm the relation between NS and negative evaluations in other sensory dimensions^49^,^50^. According to the findings of this study, NS is selectively associated with alteration of neuronal processing of noise, but not of intensity, location, frequency or rhythm contour changes. Therefore, NS is probably specific to the acoustic properties of noise, and is related to their neuronal processing. How this altered noise-specific defect of the auditory system is linked to negative reactions towards sounds is to be determined by future research.

Research on hearing loss in older adults and young adults suggests that hearing deficits require more resources for sound processing, e.g., during segregation of a signal from noise. According to the “effortfulness hypothesis”, a decline in comprehension and recall of speech in noise in such subjects is related to the greater demand that these individuals allocate to the auditory processing, producing decrements to cognitive functions. According to Reber and his co-authors^51^, processing fluency is linked to affective responses in a way that high fluency is associated with positive evaluations. For instance, images that are primed with a matching contour are not only recognized faster, indicating a higher processing fluency, but are also liked more than the images preceded by an unmatching prime^52^. It was shown in an EEG study that noise sensitive individuals are more aroused during task performance in a noisy background than in silence, as was indicated by an increased power of high frequency (gamma) bands, whereas, non-sensitive subjects remained at intermediate levels during both conditions^53^. Moreover, they were aroused by noise regardless of the magnitude of sound annoyance, while non-sensitive participants were aroused only by the presence of most annoying sounds, indicating a difference in central auditory processing^54^. That could relate to an extra effort that noise sensitives need to put in order to perceive sounds. We may speculate that noise sensitive individuals struggle with predicting the noisy soundscape of the daily environment, which manifests itself behaviorally as negative judgments towards sounds and noise annoyance.

The neuronal mechanisms of sound processing may be the key to understanding the origin of NS. However, we do not imply that functional alternations in the auditory cortex are causal to NS. It has been shown that subjects continuously exposed to noisy auditory environments, but without peripheral damage, may develop subpathological changes in cortical responses to sounds, especially to noisy ones. For instance, a pioneering study showed that in control subjects the MMN was larger to non-speech than speech sounds while it did not differ between the sound types in the noise-exposed subjects. In exposed workers, the MMN to speech sounds was lateralized to the right hemisphere, while in control subjects it was left-hemisphere predominant^4^. Thus, long-term noise exposure altered the strength and the hemispheric organization of speech-sound discrimination and decreased the speed of sound-change processing^55^. It is possible that the functional changes in the auditory system observed here in noise sensitive individuals would result from the susceptibility of their central auditory system to detrimental noise effects.

In this study, we did not control for individual intensity levels at which the experimental paradigm was delivered. Instead, we controlled the individually perceived comfortable loudness levels. We argue that the participants did not exhibit apparent differences in preferred sound intensity that would strongly influence the results. If we suppose, however, that subjects with high NS could prefer lower intensities of stimulus presentation that would more likely influence the MMN response to the intensity deviant than the MMN in general. It has previously been observed that higher sound intensity evokes stronger MMN to intensity decrement^56^, but that MMN to frequency change is independent of the intensity of stimuli presentation^56^,^57^. Accordingly, if the MMN attenuation in high NS group was due to a lower energy of sound presentation, we would expect to find a reduced MMN response to the intensity change rather than to the features that are based on spectral auditory information (pitch, noise and pitch slide). Contrary to this expectation, the MMN to the intensity change was not different between the NS groups. Thus, we think that our results were not affected by the differences in the loudness at which the stimuli were presented to the individual subjects.

Even though there is still no clear answer to the underlying mechanisms of NS, it is evident that the neurophysiological approach has a potential to advance the understanding of this phenomenon. Discerning the role of the central nervous system in NS will bear implications for health care: it will help in taking appropriate steps towards decreasing the health risks in the susceptible population, and in opening new avenues for individualized compensatory strategies against annoyance from environmental noise. We hope that our findings will encourage researchers to study NS with objective measures in order to achieve these goals.

## Methods

### Participants

Originally, 71 healthy subjects participated in the study. EEG data of five subjects were discarded due to low data quality. Hence, the final data set consisted of 71 MEG recordings (34 men, 37 women, age range 19–51, M = 28.48) and 66 EEG recordings (32 men, 34 women, age range 19–51, M = 28.67). None of the participants reported neurological or hearing dysfunctions.

All participants signed an informed consent on arrival to the laboratory and received compensation for their time. All experimental procedures for this study, included in the broad research protocol termed “Tunteet”, were approved by the Coordinating Ethics Committee of the Hospital District of Helsinki and Uusimaa (the approval number 315/13/03/00/11, obtained on March the 11^th^, 2012). All procedures were conducted in agreement with the ethical principles of Declaration of Helsinki.

### Questionnaires

NS was studied using the Weinstein’s Noise Sensitivity Scale^58^. It consists of 21 statements to rank an agreement with on a 6-point scale with endpoints at 1 (“agree strongly”) and 6 (“disagree strongly”), of which 14 statements are reverse-scored before responses are summed. The total sum represents NS. A higher number corresponds to greater NS and vice versa.

This questionnaire was incorporated into the internet-based Helsinki Inventory of Music and Affective Behaviors^59^ (HIMAB). The HIMAB also included questions regarding subjects’ musical background (e.g., years of musical training that we used in this study to control for musical experience). Subjects were provided the link to the online form and completed it at home. In a case of any questions about filling the form, they could contact a research assistant via phone at any time.

In the laboratory subjects completed Hospital Anxiety and Depression Scale^60^ (HADS-A) questionnaire. The depression subscale was used to measure levels of depression.

### Stimuli

**Figure 1:**
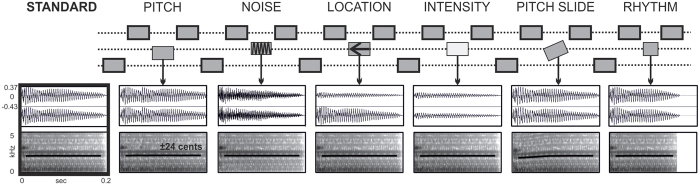
Experimental stimuli. The first row illustrates a piano tone sequence organized in patterns of four, where the third tone is a deviant (thin outline) and the other three are standards (thick outline). The presentation of deviant types was randomized. The dotted lines represent frequencies that correspond to notes of a tonic chord. The key was changed periodically in a pseudo-random order. Each tone was 200 ms in duration with an interstimulus interval (ISI) of 5 ms. The rows below represent an example sound waveform (upper) and a spectrogram (lower) of a standard and deviants. The thick black lines on the spectrograms represent the base frequency.

**Figure 2:**
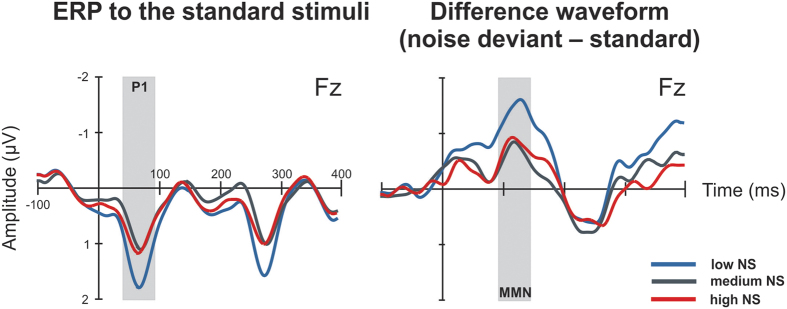
Group-averaged ERPs and difference waveforms. ERPs to the standard stimuli are on the left and the difference waveforms obtained by subtracting the ERP to the standard from the ERP to the noise deviant are on the right. P1 and MMN waveforms are indicated on the figure. NS = noise sensitivity.

**Figure 3:**
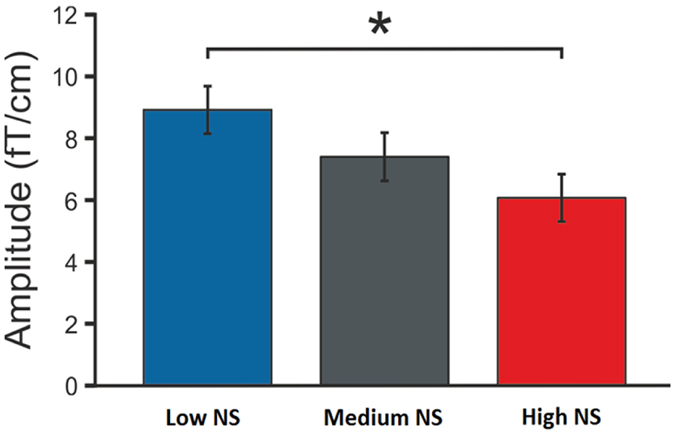
Mean amplitudes of magnetic MMN to the noise deviant in low, medium and high NS groups across all ROIs. The asterisk indicates a significant difference (*P* = 0.007). NS = noise sensitivity.

Stimuli were synthesized piano tones of different pitch. The duration of a single tone was 200 ms with 5ms of fall and rise times, and with ISI of 5ms. They were arranged in patterns of four as in a chord arpeggio (root–fifth–third–fifth, Fig. 1). The resulting musical sequence is an accompaniment of frequent occurrence in Western music. The 3rd tone of each pattern was a deviant of one of the six types: noise, pitch, location, intensity, pitch slide, and rhythm. The key was transposed after each of deviations was played in the current key once. All 24 possible keys were used. The order of deviations and keys were pseudo-randomized. Sound waveforms and spectrograms of the stimuli are visualized in Fig. 1. For the noise deviant, the timbre was modified in Adobe Audition by applying the ‘old-time radio’ effect. As compared to the standard sound, it was characterized by increased zero-crossing rate (t = −6.55, p < 0.0001). The feature was extracted with MIRToolbox in MATLAB environment^61^. The zero-crossing rate is a basic sound measure that reflects the number of sign changes of the waveform. This feature defines sound noisiness. The rhythm deviant was a shortened note of 160 ms. Consequently the following tone appeared earlier producing a change in a rhythmic contour. Hence, in the patterns with the rhythm deviation, the fourth tone of a pattern was exceptionally analyzed as a deviant. The intensity deviant was a 6 dB intensity reduction. The pitch deviant was tuned 24 cents up in a major mode and down in a minor mode. The slide deviant was a continuous modulation of sound frequency from two semitones below up to the standard. The location deviant was made by decreasing the amplitude of the right channel to 10 dB, perceptually resulting in a sound coming slightly from the left. Detailed description of the deviants were in previous studies with fast musical multifeature MMN paradigm^31^,^32^,^33^. An audio sample of the no-standard multifeature MMN paradigm can be found in the Supplementary information.

### Data acquisition and preprocessing

The recording was done in an electrically and magnetically shielded room (ETS-Lindgren Euroshield, Eura, Finland) at the Biomag Laboratory of the Helsinki University Central Hospital. Participants were comfortably seated in a chair with their head placed inside a helmet-like space of MEG machine. The sound was delivered by a pair of pneumatic headphones. During a sound-check prior to the measurement, the loudness of the stimuli was adjusted to a level that was comfortable to the individual. The chosen levels were very similar among individuals. The subjects were instructed to remain still during the recordings. During the measurement, subjects were watching a silenced movie of their own choice with subtitles.

The data were recorded with a 306-channel Vectorview™ whole head MEG device (ElektaNeuromag^®^, Elekta Oy, Helsinki, Finland) and a compatible EEG system. The MEG device had 102 pairs of planar gradiometers and 102 magnetometers. A 64-channel EEG electrode cap was connected to an amplifier for a simultaneous EEG and MEG recording. The reference electrode was attached to the tip of the nose and the ground electrode to the right cheek. Vertical eye movements and blinks were measured with two electrodes placed above the left eyebrow and on the cheek below the left eye. Horizontal eye movements were measured at the temples close to external eye corners. Additionally, four head position indicator coils were placed on top of the EEG cap and located respectively to nasion and preauricular anatomical landmarks by Isotrack 3d digitizer (Polhemus, Colchester, VT, USA). MEG/EEG data were recorded with a sample rate of 600 Hz.

MEG/EEG data were preprocessed with BESA Research 6.0 Software (BESA GmbH, Munich, Germany). For EEG preprocessing after a visual inspection, any channels with noisy signal were replaced by interpolating the data of the surrounding channels. The data were further processed by an automatic eye-blink correction. Any artifacts were removed automatically using ±100 μV rejection threshold for EEG data and 1200 fT/cm for MEG data. Thereafter, the EEG and MEG responses were divided into epochs time-locked to the stimulus onset using a time window of −100 to 400 ms and baseline corrected in a time window of −100 to 0 ms before the stimulus onset. The data were averaged according to the stimulus type. MEG data were additionally processed in MATLAB, where vector sums of each gradiometer pair were computed by squaring the signals and taking the square root of their sum. Next, individual areal mean curves were calculated over four areas above the left and the right temporal lobes by averaging vector sums of underlying gradiometer pairs. The areas were selected by the maximum response.

### Data analysis

For EEG data, P1 was determined and extracted from the grand averaged ERP to the standard sound at the Fz electrode. Based on visual inspection, the latency of P1 was searched as the highest point between 40 and 90 ms. The amplitude was calculated as the mean value in 40 ms time window centered at the individual peak.

Then, the ERP waveform for standard stimulus was subtracted from each deviant ERP. The MMN latencies were automatically searched in the time windows visually identified from grand-averaged difference waveforms at Fz electrode separately for each deviant: 100–250 ms for the noisy timbre, the intensity, and the rhythm deviants; 150–250 ms for the pitch deviant; 100–220 ms for the slide deviant; 70–150 ms for the location deviant. The mean amplitudes were extracted from frontal and central electrodes (Fz, F3, F4, C3, C4) as the averaged values over ±20 ms time window around the peak latency identified from Fz electrode. The polarity reversal of MMN was evaluated at TP9 and TP10 channels since mastoid electrodes were not provided in the EEG system.

For MEG data, the amplitudes and latencies were automatically extracted from the averaged subtracted areal mean curves similarly to the EEG data.

### Statistical analysis

In order to test whether NS is related to neural sound processing and feature discrimination, we employed analyses of variance (ANOVA). For that, we divided subjects into three groups according to their NS (low, medium and high) using a tertile split. As observed in the literature, musical training affects brain functions related to sound processing especially to musical sounds^62^,^63^. However, NS is not associated with musicality^64^. We included years of musical training as a covariate to account for possible effects musical experience on auditory processing. In addition, the wide range of age of the subjects made us include this variable as another covariate even though age did not differ between three NS groups (F_2,70_ = 0.950, *P* = 0.35). That was to account for possible age-related decline in auditory perception which can also be reflected in MMN^65^.

Greenhouse-Geisser correction was used whenever applicable; the original degrees of freedom and corrected *P* values were reported.

## Additional Information

**How to cite this article**: Kliuchko, M. *et al*. A window into the brain mechanisms associated with noise sensitivity. *Sci. Rep.* **6**, 39236; doi: 10.1038/srep39236 (2016).

**Publisher’s note:** Springer Nature remains neutral with regard to jurisdictional claims in published maps and institutional affiliations.

## Supplementary Material

Supplementary Tables and Figures

Supplementary Audio 1

Supplementary Audio 2

Supplementary Audio 3

Supplementary Audio 4

Supplementary Audio 5

Supplementary Audio 6

Supplementary Audio 7

Supplementary Audio 8

## Figures and Tables

**Table 1:** Amplitudes and latencies of P1 and MMN to different sound feature deviations recorded at Fz electrode.

	Mean Amplitude (mV)	SD	Mean Latency (ms)	SD
P1	1.2	0.7	67	10
Pitch-MMN	−1.3	1.0	198	21
Noise-MMN	−1.3	1.1	138	27
Location-MMN	−2.5	1.6	120	12
Intensity-MMN	−1.0	1.0	158	31
Slide-MMN	−1.7	1.1	186	21
Rhythm-MMN	−1.5	1.2	155	23
