# Supplementary material for: A window into the brain mechanisms associated with noise sensitivity
Source: Sci Rep. 2016 Dec 15;6:39236. doi: 10.1038/srep39236 (PMC5157031; doi:10.1038/srep39236)
Supplement: Supplementary Tables and Figures [file srep39236-s1.pdf]

A window into the brain mechanisms associated with noise sensitivity

Marina Kliuchko*, Marja Heinonen-Guzejev, Peter Vuust, Mari Tervaniemi & Elvira Brattico

Supplementary information

	Mean Amplitude	*SD*	*t*	*dF*	*P*	Mean Latency	*SD*
Pitch-MMN							
Fz	-1.4	1.1	-12.8	65	0.000	199	21
TP9	0.5	1.0	4.9	65	0.000		
TP10	0.7	1.0	7.6	65	0.000		
Noise-MMN							
Fz	-1.3	1.1	-11.4	65	0.000	140	27
TP9	0.2	0.8	2.8	65	0.007		
TP10	0.5	0.9	5.3	65	0.000		
Location-MMN							
Fz	-2.5	1.5	-17.2	65	0.000	120	12
TP9	0.7	1.0	6.8	65	0.000		
TP10	0.9	1.1	8.5	65	0.000		
Intensity-MMN							
Fz	-1.1	1.0	-10.4	65	0.000	157	32
TP9	0.1	0.7	1.7	65	0.091		
TP10	0.3	0.7	4.2	65	0.000		
Slide-MMN							
Fz	-1.7	1.2	-14.2	65	0.000	186	22
TP9	0.7	1.2	5.2	65	0.000		
TP10	0.9	1.2	7.4	65	0.000		
Rhythm-MMN							
Fz	-1.38	1.0	-14.2	65	0.000	153	25
TP9	0.79	0.8	9.5	65	0.000		
TP10	0.78	0.9	8.4	65	0.000		

Supplementary table S1. MMN amplitudes and latencies at Fz and inferior temporal (TP9, TP10) electrodes. Significant *P*-values are shown in bold.

				ANCOVA (Main effect of Group)			Coveriate effects	
Deviation	Group	Mean Amplitude*	SD	**F**	**P**	**η**p2	**P** (Years of Musical Training)	**P** (Age)
Pitch	Low NS	-1.64	0.21	1.76	0.180	0.055	0.004	0.262
	Medium NS	-1.16	0.21					
	High NS	-1.15	0.22					
Noise	Low NS	-1.93	0.22	6.14	0.004	0.168	0.418	0.059
	Medium NS	-0.97	0.21					
	High NS	-1.02	0.22					
Location	Low NS	-3.06	0.33	3.83	0.027	0.111	0.199	0.233
	Medium NS	-1.84	0.32					
	High NS	-2.70	0.34					
Intensity	Low NS	-1.41	0.20	2.56	0.086	0.077	0.071	0.041
	Medium NS	-0.84	0.19					
	High NS	-0.89	0.21					
Slide	Low NS	-1.99	0.23	2.12	0.128	0.065	0.044	0.272
	Medium NS	-1.34	0.22					
	High NS	-1.69	0.23					
Rhythm	Low NS	-1.63	0.21	0.50	0.611	0.016	0.081	0.095
	Medium NS	-1.35	0.20					
	High NS	-1.44	0.21					

*Mean values are adjusted for the effect of Years of Musical Training and Age covariates.

Supplementary Table S2. Results of separate ANCOVAs testing MMN amplitude differences between noise sensitivity (NS) groups.

				ANCOVA (Main effect of Group)			Coveriate effects	
Deviation	Group	Mean Amplitude*	SD	**F**	**P**	**η**p2	**P** (Years of Musical Training)	**P** (Age)
Pitch	Low NS	10.69	1.18	3.04	0.055	0.084	0.012	0.991
	Medium NS	8.60	1.18					
	High NS	6.55	1.16					
Noise	Low NS	8.92	0.74	3.82	0.027	0.104	0.237	0.058
	Medium NS	7.46	0.74					
	High NS	6.00	0.73					
Location	Low NS	14.71	1.48	1.65	0.201	0.047	0.383	0.002
	Medium NS	13.16	1.48					
	High NS	10.89	1.45					
Intensity	Low NS	6.82	0.62	2.02	0.140	0.058	0.259	0.048
	Medium NS	5.80	0.62					
	High NS	5.06	0.60					
Slide	Low NS	13.78	1.10	2.99	0.057	0.083	0.000	0.013
	Medium NS	13.85	1.09					
	High NS	10.50	1.07					
Rhythm	Low NS	9.16	1.06	0.004	0.996	>0.0001	0.015	0.534
	Medium NS	9.21	1.06					
	High NS	9.09	1.04					

* Mean values are adjusted for the effect of Years of Musical Training and Age covariates.

Supplementary Table S3. Results of separate ANCOVAs testing MMNm amplitudfe differences between noise sensitivity (NS) groups.

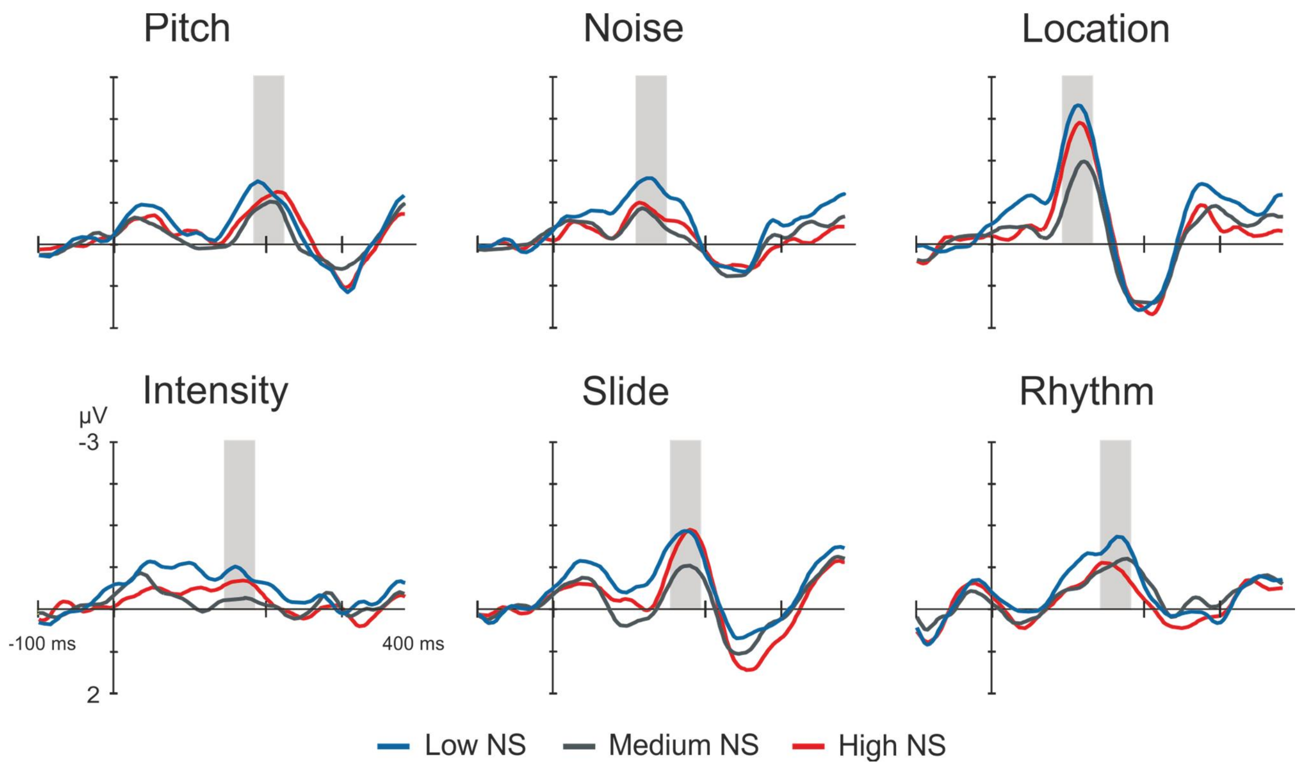

Supplementary Figure S1. Difference waveforms for six types of deviants for MMN responses obtained from Fz electrode.

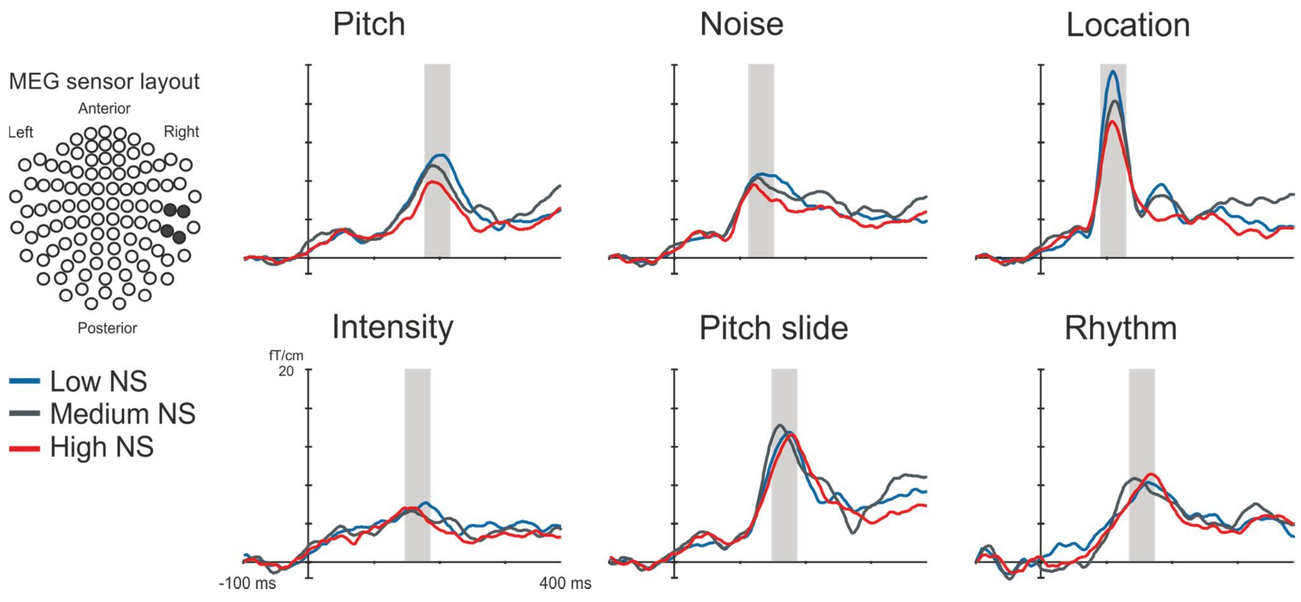

Supplementary Figure S2. Mean difference waveforms for six types of deviants. The illustrated responses were obtained from a group of sensors over the right hemisphere with a largest response.

Supplementary Audio **1** (Caption)

An excerpt of the no-standard musical multifeature mismatch negativity paradigm.

Supplementary Audio **2** (Caption)

A standard sound.

Supplementary Audio **3** (Caption)

A pitch deviant.

Supplementary Audio **4** (Caption)

A noise deviant.

Supplementary Audio **5** (Caption)

A location deviant.

Supplementary Audio **6** (Caption)

An intensity deviant.

Supplementary Audio **7** (Caption)

A pitch slide deviant.

Supplementary Audio **8** (Caption)

A rhythm deviant.
